# Le Fort I maxillary osteotomy in a Jehovah’s Witness patient: strategies for minimizing blood loss and maximizing safety

**DOI:** 10.1186/s40902-022-00338-6

**Published:** 2022-03-02

**Authors:** Michaela K. O’Connor, Elisa Emanuelli, Ravi K. Garg

**Affiliations:** 1 University of Kansas School of Medicine, Kansas City, KS USA; 2 Department of Plastic Surgery, University of Kansas Medical Center, Kansas City, KS USA

**Keywords:** Blood Transfusion/ethics*, Jehovah’s Witnesses*, Osteotomy, Le Fort, Blood loss, Surgical*, Orthognathic Surgical Procedures/methods*

## Abstract

**Background:**

The Watch Tower Society, the main governing organization of the Jehovah’s Witness (JW) faith, introduced the doctrine to refuse blood in 1945 and has been enforcing it since 1961. A member can be expelled for accepting prohibited blood components. Many reconstructive surgeries place patients at an increased risk for blood loss. There have been attempts at reducing the rate of transfusions in craniofacial surgery, even in patients not opposed to it.

**Presentation:**

A 15-year-old female patient, who refused blood transfusion due to her faith, presented with a class III malocclusion, transverse maxillary constriction, and a lateral open bite. Surgery was deferred until the patient reached 18 years of age and had undergone preoperative orthodontics. A two-piece Le Fort I osteotomy was performed. Erythropoietin, oral iron, and tranexamic acid were used to minimize intraoperative blood loss.

**Conclusion:**

Here we discuss preoperative and intraoperative management strategies to ensure a transfusion-free environment for patients with religious objections to blood transfusions undergoing operations with increased bleeding risk.

## Background

The Watch Tower Society, the main governing organization of the Jehovah’s Witness (JW) faith, introduced the doctrine to refuse blood in 1945 and has been enforcing it since 1961 [11]. A member can be expelled for accepting prohibited blood components [11]. Refusal of blood products is based on several Bible passages, including Genesis 9:4; Leviticus 17:10; Deuteronomy 12:23; Acts 15:28, 29; and Leviticus 17:14 [19]. Patients of the JW faith generally refuse whole blood, platelets, fresh frozen plasma, cryoprecipitates, granulocytes, and autologous blood that has been removed from their body. Albumin, immunoglobulins, factor concentrate, organ, and tissue transplants are left to the discretion of the individual [4].

Many reconstructive surgeries place patients at an increased risk for blood loss. Previous studies show the blood loss during a Le Fort I osteotomy ranges from 50 to 3400 ml [10, 15–17]. Around 2% of patients undergoing a Le Fort I osteotomy require blood transfusion, although this usually occurs when another procedure such as mandibular surgery or bone grafting is simultaneously performed [6, 10].

## Case presentation

A 15-year-old otherwise healthy female presented to the clinic for orthognathic evaluation. She reported difficulty chewing, would bite her tongue, and experienced some TMJ clicking and popping. She and her family are Jehovah’s witnesses and object to receiving blood products. It was decided to defer surgery until she reached adulthood so she could legally make her own decisions about blood transfusion.

The patient returned 2 years later, at the age of 17, for re-evaluation. She continued to report difficulty with mastication, pain with eating hard foods, and occasional tongue biting. On physical exam, she had class III malocclusion, transverse maxillary constriction, and a lateral open bite. She was not experiencing any nasal or upper airway obstruction.

She returned 9 months later, at age 18. As an adult, she could consent for surgery and legally refuse blood transfusions. Blood management strategies were discussed with the patient. The patient agreed to oral iron supplementation and erythropoietin (EPO) preoperatively. The patient was also willing to use a cell saver if the circuit was connected to her the entire time. The surgeon and patient discussed the possible need to abort surgery and complete it in stages if significant bleeding occurred with possible need for interventional radiology to embolize branches of the external carotid system. The options for a single versus double jaw surgery were reviewed with the patient. It was determined that adequate occlusion and optimal safety could be achieved with a two-piece Le Fort I osteotomy. Virtual surgical planning was used.

**Fig. 1:**
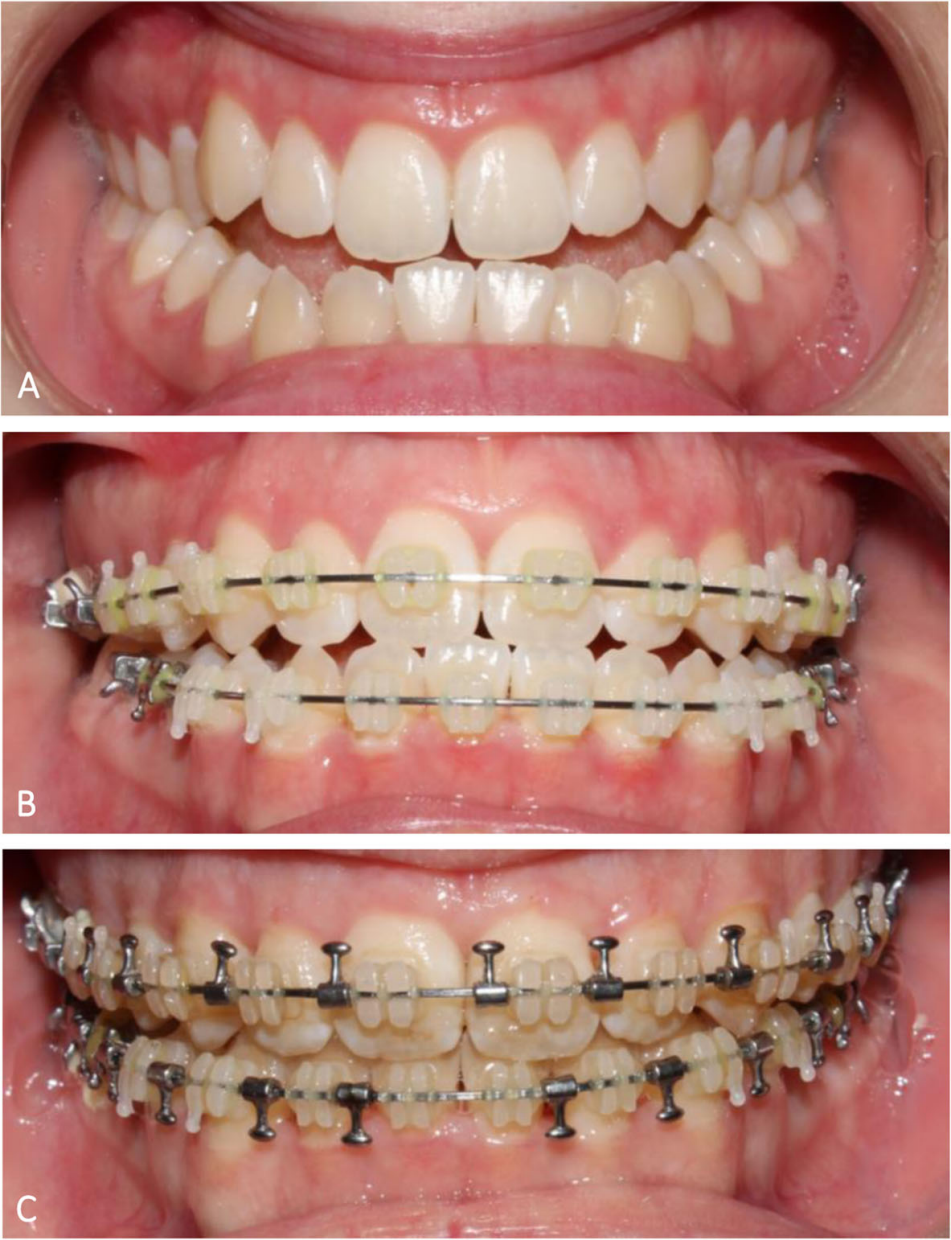
Occlusion photos from **A** before orthodontics, **B** preoperative, and **C** 6 weeks postoperative

Prior to surgery, anesthesiology was consulted to plan safety measures in accordance with the patient’s mandates. A baseline CBC showed a hemoglobin of 13.1 g/dl. She was started on iron and EPO. On the day of surgery, her hemoglobin was 16.8 g/dl. She underwent an uncomplicated surgery with a two-piece Le Fort I maxillary osteotomy and bone graft. Anesthesia was induced with sevoflurane, midazolam, fentanyl, propofol, and rocuronium. It was then maintained with sevoflurane, ketamine, propofol, and remifentanil. Intraoperatively, she received a 1000-mg tranexamic acid bolus followed by a 1-mg/kg/h drip. A cell saver was not required but was immediately available. The estimated blood loss was 100 ml. Her hemoglobin on postoperative day 1 was 15.5 g/dl. She was discharged home that day. She had no complications and at follow-up had a stable occlusion and improved profile (Fig. 1).

## Discussion

There are many known strategies to improve outcomes and decrease risk in patients who refuse blood products and are undergoing operations with risk of blood loss (Table 1). Jehovah’s Witnesses have a worldwide network of Hospital Liaison Committees that can assist physicians and provide information on clinical strategies to avoid blood transfusions.

**Table 1:** Strategies for minimizing blood loss

	Purpose/goal
**Preoperative**	
Erythropoiesis-stimulating agent^a^ (Epoetin alfa)	Optimize preoperative red cell mass [21]
Oral or IV iron^a^	Ensure adequate iron stores to support erythropoiesis
**Intraoperative**	
Minimize phlebotomy, use small-volume pediatric blood tubes if blood tests are needed	Reduce iatrogenic blood loss through ordering appropriate blood draws
Hypotensive anesthesia^a^	Lowers hydrostatic pressure of damaged vessels. Statistically significant reduction in blood loss [3, 8]
Normovolemic hemodilution	Reduce blood loss and prevents coagulopathy [2]
Intraoperative cell salvage^b^	Collect and re-infuse lost blood; may be acceptable to JW patients if the blood remains in continuity with their circulation [1]
Perfluorocarbons	Improvement of microcirculation, transport of nitric oxide, and clearance of lactates; questionable ability to deliver adequate oxygen at normal partial pressure [18]
Tranexamic acid^a^	Impair endogenous fibrinolysis [9]; shown to reduce blood loss and/or transfusion in craniofacial and orthognathic surgery [12]
Embolization^b^	Decrease blood flow through hemorrhaging blood vessels; most often performed by interventional radiology

^a^Used in this case

^b^Discussed with the patient but not ultimately needed in this case

There have been previous case reports of Le Fort I osteotomies performed in JW patients [7, 15]. One of these cases was similar to our case consisting of a teenage female undergoing a two-piece Le Fort I maxillary osteotomy to correct a class III malocclusion. This previous case was a double jaw surgery that included a mandibular setback in addition to the Le Fort I maxillary osteotomy. The patient received both EPO and iron and was set up with hemodilution and a cell saver that was kept in continuity with the patient’s circulatory system. Unlike our patient, this patient was not reported to receive tranexamic acid (TXA). This may account for this patient’s significantly higher blood loss, estimated at 600 ml compared to the 100-ml blood loss seen in our patient who did receive a TXA bolus and infusion during surgery [15].

TXA is an antifibrinolytic drug that competitively inhibits plasminogen and plasmin and blocks inflammatory cell activation and chemotaxis, leukotriene synthesis, and proinflammatory gene expression [12]. While TXA is largely well tolerated, gastrointestinal distress, allergic skin reactions, and visual disturbances can occur [13]. In addition, TXA carries a dose-dependent risk for seizures [13]. While there is concern that TXA increases thromboembolic risk, multiple large meta-analyses have found no evidence to support this [5, 12–14].

A case series out of Korea reported their algorithm and approach to patients undergoing orthognathic surgery who refuse blood transfusions. All patients receive preoperative EPO and IV iron. Those with a preoperative hemoglobin less than 10 g/dl receive three doses, while those that have a higher hemoglobin receive one dose. Intraoperatively, they use acute normovolemic hemodilution and hypotensive anesthesia. Postoperative day 1, they give EPO and IV iron again. If the hemoglobin is below 10 on postoperative day 3, patients receive three more doses. No patient required a transfusion, and all were discharged without complications [7].

There have been attempts at reducing the rate of transfusions in craniofacial surgery, even in patients not opposed to it. A recent study of infants undergoing craniosynostosis repair, a surgery historically associated with 100% transfusion rates, showed that transfusion rates were reduced by 92% with the implementation of a blood conservation protocol. The protocol included preoperative ferrous sulfate and EPO, infiltration of local anesthetic with epinephrine, PlasmaBlade incision and subgaleal dissection, hypervolemic hemodilution, and tranexamic acid [20]. These are strategies that may be considered in other craniofacial operations as well for patients with objections to receiving blood products.

## Conclusion

Le Fort I osteotomies can cause significant bleeding on rare occasion. Patients who refuse blood transfusions undergoing this procedure require additional counseling and preoperative planning. It is important to have open discussions about what products or procedures are acceptable to the patient, in advance of surgery. This case utilized iron supplementation and EPO preoperatively and tranexamic acid and hypotensive anesthesia to ensure a good surgical outcome, without sacrificing safety, in a Jehovah’s Witness patient.

## Data Availability

Data sharing is not applicable to this article as no datasets were generated or analyzed during the current study.

## Abbreviations

JW: Jehovah’s Witness (JW)
EPO: Erythropoietin
